# Evaluation of the effect of locoregional treatment on metabolic liver function in hepatocellular carcinoma using ^18^F-FDGal PET/CT

**DOI:** 10.1186/s13550-025-01285-9

**Published:** 2025-07-16

**Authors:** Mona Kjærbøl Kristiansen, Kirstine Petrea Bak-Fredslund, Stine Kramer, Gerda Elisabeth Villadsen, Michael Sørensen

**Affiliations:** 1 Department of Hepatology & Gastroenterology, Aarhus University Hospital, Palle Juul- Jensens Boulevard 35, Aarhus N, DK-8200 Denmark; 2 Department of Gastroenterology & Hepatology, Aalborg University Hospital, Mølleparkvej 4, Aalborg, DK-9000 Denmark; 3 Department of Nuclear Medicine & PET Centre, Aarhus University Hospital, Palle Juul- Jensens Boulevard 165, Aarhus N, DK-8200 Denmark; 4 Department of Internal Medicine, Viborg Regional Hospital, Heibergs Alle 5A, Viborg, DK-8800 Denmark

**Keywords:** Hepatocellular carcinoma, Liver imaging, Liver regeneration, Metabolic liver function, PET/CT

## Abstract

**Background:**

The effect of different locoregional treatments for hepatocellular carcinoma (HCC) on metabolic liver function is largely unknown. This information is crucial, particularly for patients with cirrhosis. We applied [^18^F]-fluoro-2-deoxy-*D*-galactose (^18^F-FDGal) positron emission tomography (PET) to determine the contribution of large HCCs to total metabolic liver function and the changes in metabolic liver function post-treatment.

**Results:**

We included 29 patients with HCC treated with resection (*n* = 8), radiofrequency ablation (RFA) (*n* = 8), transarterial chemoembolization (TACE) (*n* = 9), and selective internal radiation therapy (SIRT) (*n* = 4). In patients with HCCs > 3 cm, the liver’s total metabolic activity was significantly higher when including the metabolically active tumor areas compared to when the tumor was excluded (*p* = 0.0002). The median percent change in mean metabolic activity in the liver after locoregional treatment was 5.1% in patients without cirrhosis as compared to -6.0% in patients with cirrhosis (*p* = 0.05). The distribution of cirrhosis (*n* = 15 in total) among treatment groups was uneven. After treatment, seven of eight patients who underwent resection showed increased or stable mean metabolic liver function, while responses for those treated with RFA, TACE, or SIRT were mixed. Changes in mean metabolic liver function and liver volume did not correlate.

**Conclusions:**

HCCs > 3 cm contributed substantially to the liver’s galactose metabolism, suggesting that this would also apply to other substrates used for measuring metabolic liver function. Changes in metabolic capacity following treatment depend on cirrhosis status and type of treatment. Changes in functional liver volume do not necessarily reflect total metabolic capacity. The study underlines the power of imaging-based quantification of metabolic liver function.

**Supplementary Information:**

The online version contains supplementary material available at 10.1186/s13550-025-01285-9.

## Introduction

Predicting post-treatment metabolic liver function in patients treated for hepatocellular carcinoma (HCC) is crucial, particularly in those with cirrhosis [1]. Various methods such as the systemic indocyanine clearance (ICG) tests and functional magnetic resonance imaging (MRI) have been investigated for assessing pre-treatment liver function in surgical settings [2–4], but no single evaluation tool is universally recommended [5]. Published studies evaluating liver function before and after locoregional treatment for HCC with radiofrequency ablation (RFA), transarterial chemoembolization (TACE), and selective internal radiation therapy (SIRT) are virtually non-existent.

Positron emission tomography (PET)/CT using the tracer 2-^18^F-fluoro-2-deoxy-D-galactose (^18^F-FDGal), which is based on the validated galactose elimination capacity test [6–11] has proved promising for evaluating total and regional liver metabolism in terms of standardized uptake value [12]. At our center, we have years of experience with ^18^F-FDGal PET/CT both for staging HCC [13, 14] and for quantification of metabolic liver function [12, 15, 16]. Whereas several contraindications limit the broad use of functional MRI, ^18^F-FDGal PET/CT do not have any contraindications and can thus be offered to all eligible patients. Moreover, ^18^F-FDGal PET/CT enables quantification of not only the overall function of the liver, such as the ICG clearance tests do, but also liver heterogeneity which is pronounced in cirrhosis [15] and non-alcoholic steatohepatitis (NASH) [16] as well as regional changes after e.g. stereotactic body radiation therapy (SBRT) of liver tumors [17, 18]. Finally, the hepatic metabolism of ^18^F-FDGal is purely determined by enzymatic capacity, true metabolic function, and not changes in flow or hormones [19].

In this study, we thus used ^18^F-FDGal PET/CT to explore the contribution of HCCs to the total hepatic metabolism of galactose and to investigate the liver’s regenerative potential to locoregional treatments for HCC, including surgical resection, RFA, TACE, and SIRT.

### Methods

#### Study population and design

Eligible patients were identified at the multidisciplinary tumor board evaluating patients with HCC at Aarhus University Hospital, Denmark. Criteria for inclusion were age above 18 years and planned locoregional treatment for HCC with either resection, RFA, TACE, or SIRT.

#### PET/CT scans

The ^18^F-FDGal PET/CT scans were conducted after a six-hour fast as previously described [12, 13]. In short, a low-dose CT scan was performed first and used for attenuation correction of the PET data and anatomical co-registration of the PET images. A bolus of ^18^F-FDGal (median 102 MBq; range 62–117 MBq) was injected intravenously. For dynamic scans, the patients were scanned either from 0 to 20 min or 10–20 min after the injection. Static scans were performed 60 min after injection. Twenty-seven scans were conducted with both a dynamic and a static scan after the injection of ^18^F-FDGal; eleven scans were only static, whereas 20 scans were only dynamic.

The mean tissue radioactivity concentration (kBq/mL) recorded by the PET camera 60 min after the injection of ^18^F-FDGal was used for calculations. In the 20 cases with data only from dynamic scans, the functional liver volume and the mean tissue radioactivity concentration (kBq/mL) recorded by the PET camera 10–20 min after the ^18^F-FDGal injection were converted to expected values 60 min after the injection based on a positive linear relationship between the functional liver volume and the mean tissue radioactivity concentration (kBq/mL) in the liver for dynamic scans evaluated 10–20 min after injection of ^18^F-FDGal and static scans evaluated 60 min after injection of ^18^F-FDGal. The correlation was strong with a coefficient of determination (R-squared) of 0.92. (Supplementary Figs. 1 and 2 and Supplementary Table 1).

Individual functional liver volumes of interest (VOI) were created using the iso-contour tool with a tissue threshold of 6 kBq/mL [12]. The VOIs were adjusted manually for any non-liver tissue. To assess pre- and post-treatment liver metabolism, tumors with a diameter > 3 were manually removed from the VOI, including those present on the scan after treatment. Contrast-enhanced abdominal CT scans and MRI scans of the liver were used to outline the tumors, and the tumor VOIs were manually generated. The 3 cm threshold is used for staging HCC and is integral to the Milan criteria for patient eligibility in liver transplantation [20, 21]. The total radioactivity in the liver or tumor volume was calculated as the liver or tumor VOI multiplied by the mean tissue radioactivity (kBq/mL) in the liver or tumor VOI.

The total metabolic activity of the liver and in tumors > 3 cm was calculated as the percentage of the injected dose of ^18^F-FDGal accumulated in the liver or the tumor (total metabolism, %ID). The mean metabolic activity was calculated as %ID in the liver or the tumor divided by the functional volume (mean metabolism, %ID/L).

PMOD software version 4.006 (PMOD Technologies Ltd., Switzerland) was used for all image analyses.

#### Statistics

Statistical analysis was performed using Microsoft Excel (Microsoft Office Home and Student 2019, version 2106, Redmond, WA, USA) and Stata version 17 (StataCorp LLC, Texas, USA). Comparisons between groups were performed with non-parametric one-way analysis of variance, the Kruskal-Wallis test, the Wilcoxon Mann-Whitney Median test, and the Wilcoxon Signed Rank test using the exact p-value. Data are presented as median (interquartile range, iqr) unless otherwise stated.

## Results

### Baseline

**Fig. 1:**
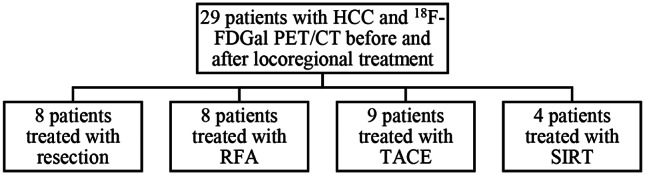
Included patients stratified by locoregional treatment. HCC, hepatocellular carcinoma, RFA, radiofrequency ablation, TACE, transarterial chemoembolization, SIRT, selective internal radiation therapy,

The study included 29 patients with a pre-treatment ^18^F-FDGal PET/CT scan a median of 12 days (range 0–40 days) before locoregional treatment for HCC and a post-treatment ^18^F-FDGal PET/CT a median of 46 days (range 31–147 days) after the locoregional treatment. All scans were conducted between November 5, 2014, and May 5, 2017, or between September 11, 2023 and September 9, 2024. Figure 1 depicts the included patients stratified by locoregional treatment.

The median age was 71 years (range 53–81 years), and 76% were male. Table 1 shows the baseline characteristics for all included patients at the time of treatment stratified by locoregional treatment.

**Table 1:** Baseline

	Resection	RFA	TACE	SIRT
**Patients, n**	8	8	9	4
**Age, years**	68.5 (62.5–75.5)	63 (59.5–72.5)	72 (71–76)	64.5 (60–69)
**Female/male, n**	0/8	4/4	1/8	2/2
**Cirrhosis, n** (%)	1 (12.5%)	6 (75%)	4 (44.4%)	4 (100%)
- Child-Pugh A5	1	4	3	3
- Child-Pugh A6	0	1	1	1
- Child-Pugh B7	0	1	0	0
**Etiology of liver disease, n**				
- Alcoholic liver disease	1	3	0	0
- Eradicated or cleared HCV	1	5	1	1
- Chronic HCV	0	0	1	1
- Other^1^	0	0	1	1
- No known liver disease	6	1	6	1
**Previous treatment for HCC, n**				
- Resection	1	0	1	1
- RFA	1	2	0	0
- SIRT	0	0	1	0
- TACE	1	0	0	0
- Systemic therapy^2^	0	0	0	2
**Time from treatment to PET/CT scan, days** ^3^	73.5 (48–104)	44.5 (36.5–99)	47 (40–50)	35 (32.5–38.5)
**Blood parameters** ^4^				
- Alanine transaminase (U/L)	23.5 (18.5–57)	30.5 (20.5–44.5)	32 (24–57)	31.5 (20.5–40)
- Bilirubin (µmol/L)	9 (6.5–11.5)	13 (9–20)	14 (10–22)	17.5 (8.5–29.5)
- Albumin (g/L)	37.5 (37–39.5)	37 (36–39)	36 (32–38)	35 (33.5–37)
- Creatinine (µmol/L)	76 (74–131)	68.5 (59–81)	77 (60–97)	58 (50–66)
- Alkaline phosphatase (U/L)	76.5 (59–111.5)	98.5 (87–138)	120 (109–180)	108.5 (94.5–137)
- AFP (ng/mL)	159.6 (18.7–894.0)	10.2 (6.0–62.7)	5.4 (0–155.4)	1280.7 (43.4 − 4377.1)

Results are presented as median (iqr) unless otherwise noted. RFA, radiofrequency ablation, TACE, transarterial chemoembolization, SIRT, selective internal radiation therapy, HCV, hepatitis C virus, PET, positron emission tomography, AFP, plasma alpha-fetoprotein.

One patient treated with RFA had both eradicated HCV and alcoholic liver disease.

^1^ Cryptogenic hepatitis and primary biliary cholangitis.

^2^ One patient had previously been treated with Sorafenib and Lenvatinib, while another patient had previously undergone chemotherapy with Capecitabine, Oxaliplatin, and Gemcitabine.

^3^ Four patients received two sessions of TACE between scan no. 1 and scan no. 2. The time was calculated from the last therapy until scan no. 2.

^4^ Data were missing for albumin for one patient treated with RFA, creatinine for one patient treated with resection and one treated with SIRT, and AFP for two patients treated with RFA and one treated with TACE.

### Tumors larger than 3 cm

Thirteen patients had tumors > 3 cm. Table 2 shows the functional volume and the metabolic activity of ^18^F-FDGal in the tumors and the liver, including and excluding the tumors on the scan before treatment. The total metabolism (%ID) was significantly higher when considering the whole liver, including the tumor, with a median of 48.1 (42.9;53.0) compared to the liver, excluding the tumor, with a median of 37.2 (33.8;42.1) (*p* = 0.0002). For further analyses, functional liver volumes refer to liver volume excluding tumors > 3 cm.

**Table 2:** Tumor and liver volume and metabolic activity in 13 patients with a tumor > 3 cm

		Functional tumor				Liver including tumor			Liver excluding tumor		
ID	Treatment	Volume (L), total	Volume (L), metabolic active	Total metabolism (%ID)	Mean metabolism (%ID /L)	Volume (L)	Total metabolism (%ID)	Mean metabolism (%ID /L)	Volume (L)	Total metabolism (%ID)	Mean metabolism (%ID /L)
1	Resection	0.083	0.083	2.0	24.3	1.826	44.1	24.2	1.743	42.1	24.2
2	Resection	0.868	0.840	14.2	16.9	2.253	51.3	22.7	1.414	37.1	26.2
3	Resection	1.141	0.833	15.8	18.9	2.319	53.0	22.8	1.484	37.2	25.0
4	Resection	0.016	0.016	0.4	23.0	1.653	42.9	26.0	1.637	42.5	26.0
5	Resection	0.065	0.043	0.6	14.2	2.053	42.4	20.7	2.010	41.8	20.8
6	TACE	0.445	0.442	7.5	17.0	2.369	56.2	23.7	1.928	48.7	25.2
7	TACE	0.040	0.040	0.7	17.5	2.387	54.7	22.9	2.347	54.0	23.0
8	TACE	0.168	0.168	3.3	19.9	1.737	50.7	29.2	1.569	47.4	30.2
9	TACE	1.529	0.943	12.7	13.5	2.462	48.1	19.5	1.519	35.4	23.3
10	TACE	1.002	1.000	26.1	26.1	2.327	59.9	25.8	1.326	33.8	25.5
11	SIRT	0.157	0.154	1.9	12.5	1.656	23.1	14.0	1.503	21.2	14.1
12	SIRT	0.396	0.327	4.8	14.7	1.470	43.6	29.7	1.143	38.8	34.0
13	SIRT	0.236	0.236	5.6	23.7	1.446	32.4	22.4	1.209	26.8	22.2

%ID, percentage of the injected dose of ^18^F-FDGal accumulated in the tumor or liver, %ID/L, percentage of the injected dose of ^18^F-FDGal accumulated in the tumor or liver per liter of tissue.

The values only include metabolically active parts of tumors with an uptake of ^18^F-FDGal of at least 6 kBq/mL. However, for the “Volume (L), total” measurement, the outer limit of the tumor was estimated using the iso-contour tool with a tissue threshold of 6 kBq/mL, but inner parts of the tumors with low metabolic activity were manually added to the volume, despite having an activity below 6 kBq/mL.

**Fig. 2:**
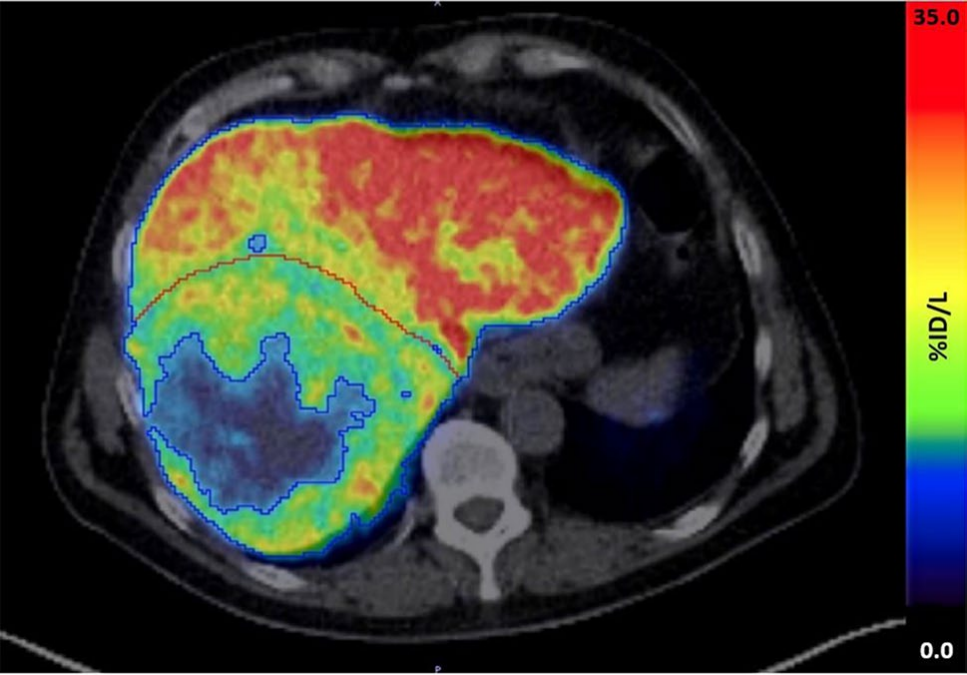
Fused axial abdominal ^18^F-FDGal PET/CT scan of a patient with a large HCC with central part with low metabolic activity. Blue line: whole liver VOI with a tissue threshold of 6 kBq/m. Red line: tumor boundary. The tumor area with low metabolic activity is included in Table 2

Figure 2 shows an ^18^F-FDGal PET/CT scan of a patient with a tumor measuring 12.5 cm in diameter with a low uptake of ^18^F-FDGal (ID 3 in Table 2).

### Impact of cirrhosis on liver ^18^F-FDGal metabolism

**Fig. 3:**
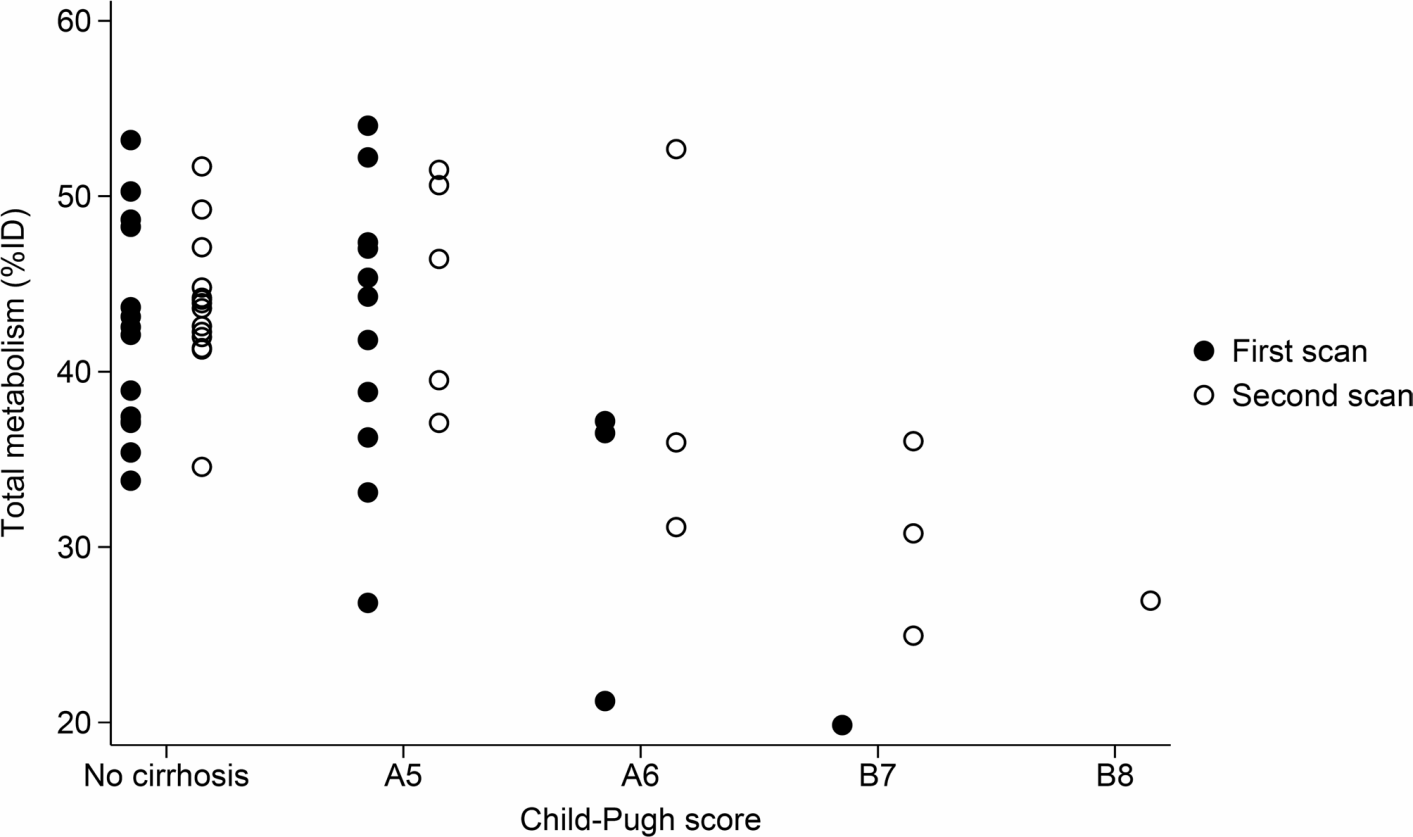
Relationship between total metabolism (%ID) and Child-Pugh score. %ID, percentage of the injected dose of ^18^F-FDGal accumulated in the liver

Of the 29 patients, 15 patients (52%) had cirrhosis, and at the time of treatment, 11 patients (73%) had a Child-Pugh score of A5, three patients (20%) of A6, and one patient (7%) of B7. Six patients had a decline in Child-Pugh score after locoregional treatment for HCC; two patients, one treated with RFA and one with SIRT, both declined from A5 to A6; one, treated with TACE, from A5 to B7; one, treated with SIRT, from A5 to B8; and two, one treated with TACE and one with SIRT, both declined from A6 to B7. Figure 3 shows the total metabolism stratified by patients without cirrhosis and the Child-Pugh score for patients with cirrhosis at the first and the second scans. The median total metabolic activity was significantly lower for patients with Child-Pugh score A6, B7, or B8 compared to patients without cirrhosis or Child-Pugh score A5 both before and after locoregional treatment (*p* < 0.05).

**Fig. 4:**
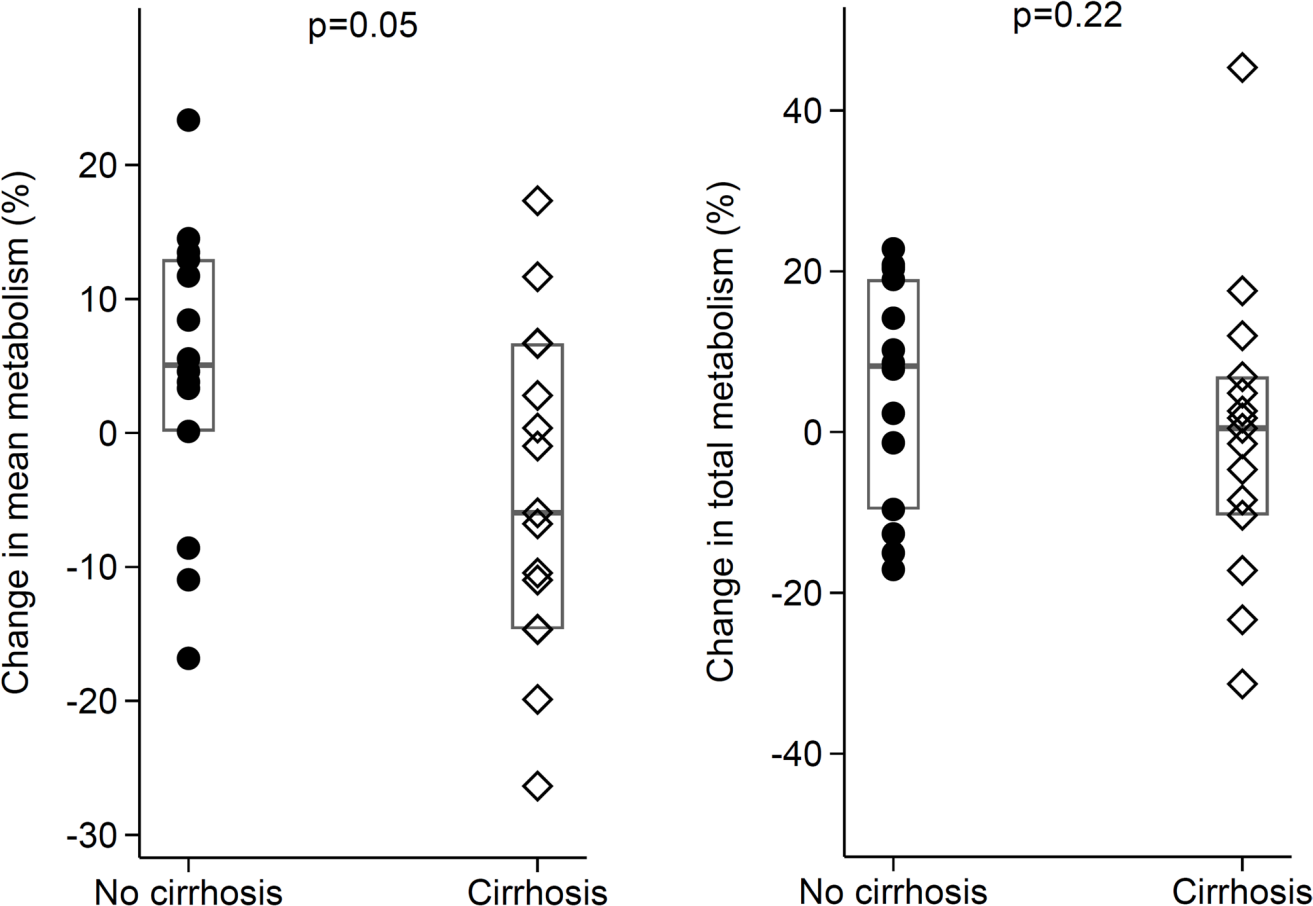
Boxplots showing relative changes in mean and total metabolism after locoregional treatment for HCC for patients with and without cirrhosis

The median percent change in the mean metabolism (%ID/L) after locoregional treatment for HCC was a 5.1% (0.12%;13.0%) increase in patients without cirrhosis, compared to a -6.0% (-14.6%;6.7%) decrease in patients with cirrhosis. This was at the statistical significance threshold for patients without cirrhosis compared to patients with cirrhosis (*p* = 0.05). The median percent change in the total metabolism was 8.2% (-9.6%;19.0%) in patients without cirrhosis and 0.47% (-10.3%;6.9%) in patients with cirrhosis (*p* = 0.22, Fig. 4).

### Liver parameters before and after local treatment

**Fig. 5:**
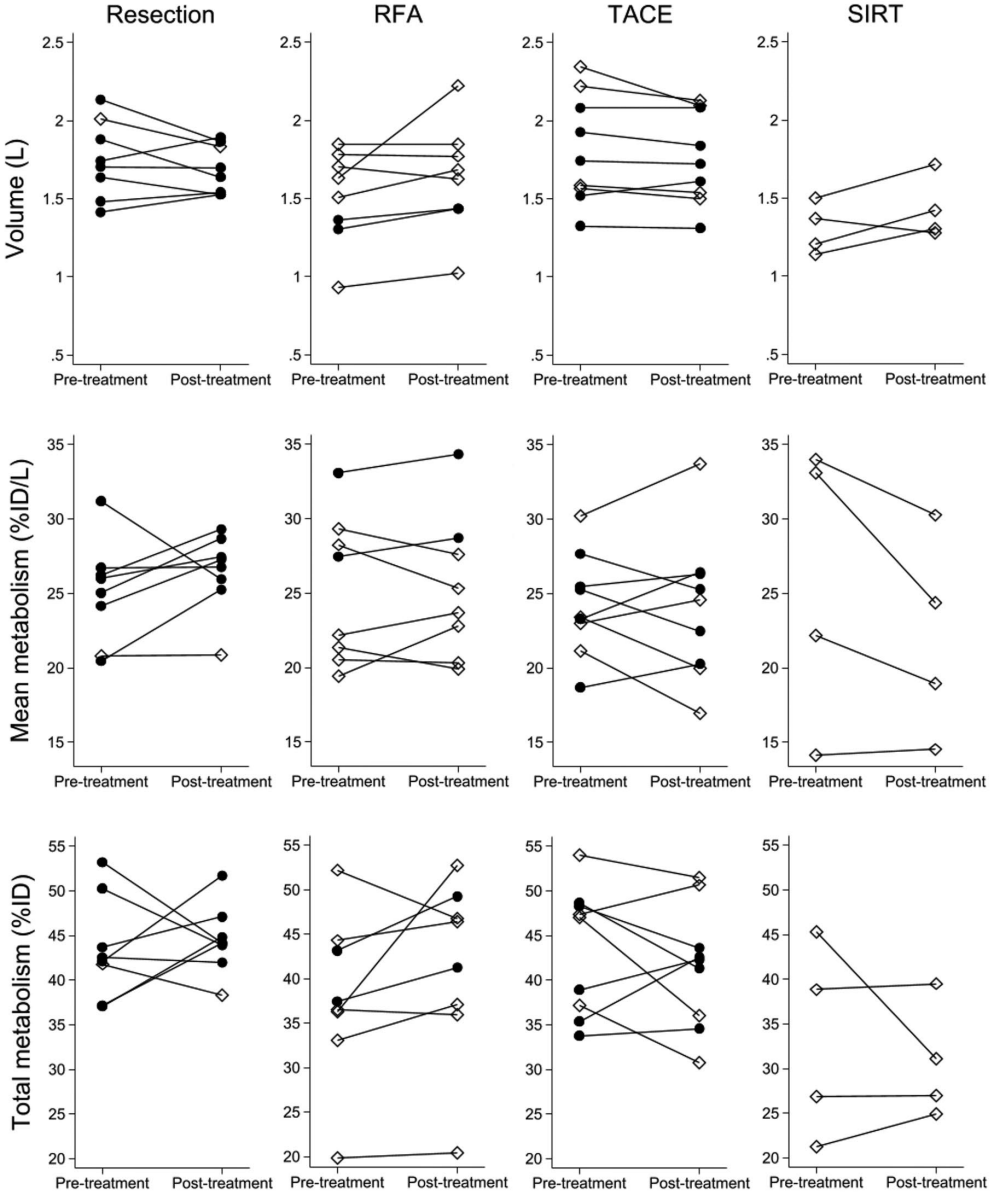
Individual changes in functional liver volume (L), mean metabolism (%ID/L), and total metabolism (%ID) stratified by treatment and +/- cirrhosis. •, No cirrhosis; ◊, cirrhosis; %ID/L, percentage of the injected dose of ^18^F-FDGal accumulated in the liver per liter of tissue, %ID, percentage of the injected dose of ^18^F-FDGal accumulated in the liver. There were no significant changes in functional liver volume, mean metabolism, or total metabolism within each treatment group

The median functional volume-to-body weight ratio before treatment was 2.08% (1.63 − 2.31%) whereas the functional volume-to-body weight ratio after treatment was 2.16% (1.92 − 2.22%). Table 3 shows the functional liver volume excluding tumors > 3 cm, mean metabolism, total metabolism, and median percent changes after locoregional treatment. There were no significant changes in any of these parameters within each treatment group. The individual paired data for each treatment modality are shown in Fig. 5, which also indicates patients with and without cirrhosis. All but one patient who underwent resection showed an increase or nearly equal mean metabolic function after treatment. The patient with decreasing mean metabolism had an inadequate partial hepatectomy 64 days prior to the first scan. The patients treated with RFA showed stable or increasing volume, except for one patient. The change in mean metabolism was mixed. One patient treated with TACE had an increase in liver volume, whereas the responses in mean and total metabolism were mixed. For patients treated with SIRT, two patients experienced an increase in liver volume, but also a decrease in mean metabolism, resulting in an almost unchanged total metabolism; one patient had an increase in liver volume and an almost steady mean metabolism; and one patient had a decrease in both liver volume and mean metabolism.

**Table 3:** ^18^F-FDGal activity in the liver

	Resection (*n* = 8)	RFA (*n* = 8)	TACE (*n* = 9)	SIRT (*n* = 4)
**Functional liver volume**				
Pre-treatment, L *	1.724 (1.560;1.946)	1.571 (1.334;1.742)	1.744 (1.569;2.081)	1.289 (1.176;1.436)
Post-treatment, L	1.670 (1.536;1.850)	1.654 (1.436;1.811)	1.725 (1.541;2.085)	1.365 (1.292;1.571)
Change in functional liver volume (%) *	-3.4 (-10.7;6.0)	7.7 (-0.2;10.8)	-3.0 (-4.3;-1.0)	14.3 (3.8;16.0)
**Mean metabolism**				
Pre-treatment, %ID/L	25.5 (22.5;26.5)	24.8 (20.9;28.8)	23.4 (23.0;25.5)	27.7 (18.1;33.5)
Post-treatment, %ID/L	27.0 (25.6;28.0)	24.5 (21.6;28.1)	24.6 (20.3;26.3)	21.7 (16.7;27.3)
Change in mean metabolism (%)	8.6 (0.24;13.7)	1.4 (-6.4;5.7)	3.3 (-11.0;8.4)	-12.8 (-20.5;-4.1)
**Total metabolism**				
Pre-treatment, %ID	42.3 (39.5;47.0)	37.0 (34.7;43.7)	47.0 (37.2;48.3)	32.8 (24.0;42.1)
Post-treatment, %ID	44.2 (42.9;45.9)	43.8 (36.5;48.0)	42.3 (36.0;43.6)	29.0 (25.9;35.3)
Change in total metabolism (%)	3.3 (-10.6;19.9)	7.5 (0.6;13.1)	-4.7 (-15.0;6.9)	1.1 (-15.4;9.7)

The results are presented as the median (iqr). RFA, radiofrequency ablation, TACE, transarterial chemoembolization, SIRT, selective internal radiation therapy, %ID/L, percentage of the injected dose of ^18^F-FDGal accumulated in the liver per liter of tissue, %ID, percentage of the injected dose of ^18^F-FDGal accumulated in the liver.

^*^ *p* < 0.05 for comparison of the four treatment groups.

There were no significant changes in functional liver volume, mean metabolism, or total metabolism within each treatment group.

Supplementary Fig. 1. Relationship between volume (mL) 10–20 min and 60 min after injection of ^18^F-FDGal.

When comparing the four treatment groups, we found a statistically significant difference between the functional liver volume at the pre-treatment scan and the change in functional liver volume (*p* = 0.03 and *p* = 0.04, respectively; Table 3). Patients treated with SIRT had a smaller mean pre-treatment functional liver volume than patients treated with resection (*p* = 0.02) and TACE (*p* = 0.01). Patients treated with RFA had a significantly higher percent change in functional liver volume than patients treated with TACE (*p* = 0.02) or resection (*p* = 0.05). There was no significant difference between the four groups for mean or total metabolism at the first or second scan.

## Discussion

In this study, we successfully used ^18^F-FDGal PET/CT to assess the contribution of hepatocellular carcinomas to the total hepatic metabolism of galactose and to evaluate the liver’s regenerative potential following locoregional treatments for HCC.

To identify the contribution of tumor activity to the overall metabolism of galactose, we analyzed 13 patients with tumors > 3 cm in diameter and found that the inclusion of these tumors in the total liver volume would wrongly overestimate the liver´s total metabolic capacity in terms of ^18^F-FDGal metabolism. This finding suggests that other substrates used to assess liver function, such as ICG, could potentially overestimate the metabolic liver function since HCC cells, like hepatocytes, take up indocyanine green [22]. These results underline the importance of using imaging-based methods to evaluate liver function, as this allows for separating the liver´s metabolic capacity from the impact of tumors and the potential metabolic heterogeneity within liver tissue, which can be pronounced in non-alcoholic steatohepatitis and cirrhosis [15, 16].

We present two factors that can influence the change in mean metabolic activity in the liver following locoregional treatment for HCC: cirrhosis status and treatment type. In line with previous studies that found a decreased regenerative potential in cirrhotic livers [2], we find that patients without cirrhosis have a higher positive mean metabolic response compared to the negative response observed in patients with cirrhosis. The results support international guidelines for liver resection which state the importance of a larger remnant liver in the case of cirrhosis [21, 23, 24].

The ability of SIRT to induce liver growth is known [25, 26], and the 2022 BCLC Guidelines for the treatment of HCC state that radiation lobectomy by SIRT may be considered to increase remnant liver volume [20]. However, SIRT is also found to have a negative effect on liver function [27], and in our study, a decline in the Child-Pugh score and mean metabolism following local treatment for HCC was mostly observed in patients treated with SIRT. Three of four patients declined the Child-Pugh scores, and all four patients either had a decreasing or, in one case, an almost steady mean metabolism. Thus, the increased functional liver volume in three patients treated with SIRT did not reflect the change in total metabolic capacity in two of them. Again, this underlines the importance of combining liver volumetry with functional measures. Using a method like ^18^F-FDGal PET/CT, both measures can be obtained in one scan.

Patients treated with RFA had an unchanged or increased functional liver volume following treatment resulting in an increase in total metabolism despite an overall steady mean metabolism. Patients treated with TACE showed an unchanged or decreased functional liver volume, whereas the change in mean metabolism varied. This is in line with recent studies that suggest that RFA is more effective than TACE for inducing growth of the future liver remnant in a two stage hepatectomy approach [28, 29]. Further, we found a tendency toward a positive response in mean metabolic activity for patients treated with resection. However, only one patient treated with resection had cirrhosis, whereas 44%, 75%, and 100% of patients treated with TACE, RFA, and SIRT had cirrhosis, respectively. Thus, a definite conclusion on the impact of treatment type is not possible from the present data, but it is worth noting that previous research suggests that liver regeneration after resection for HCC is determined by the size of the hepatectomy [30, 31]. Resection per size may be considered a larger intervention to the liver than RFA, SIRT, or TACE; thus, a higher regenerative rate may be seen after resection.

For all patients, the whole liver metabolic activity of ^18^F-FDGal was associated with cirrhosis status and Child-Pugh score, aligning with prior research indicating lower hepatic systemic clearance of ^18^F-FDGal in liver parenchyma in patients with cirrhosis compared to healthy individuals [15]. The results confirm the overall usefulness of ^18^F-FDGal PET/CT as a measure of regional and total liver function and underline that liver volume does not necessarily reflect liver function. Compared to other imaging-based tests of liver function, such as functional MRI or scintigraphy [5], which use hepatocellular uptake as a surrogate for actual liver function, ^18^F-FDGal PET/CT is a genuine metabolic measure as it quantifies the flow-independent enzymatic activity of cytosolic galactokinase [12, 19].

Our study has limitations. First, the overall median time from the locoregional treatment to the post-treatment ^18^F-FDGal PET/CT scan ranged from 31 to 147 days. However, when looking at the distribution within each treatment group, the time interval from treatment to scan was relatively consistent. After partial hepatectomy, human liver regeneration occurs within the first two weeks and is completed after three months [32] and with a median time from treatment to scan of 74 days in the present study, most patients were probably close to complete recovery in terms of both liver volume and function. The shortest interval was in the SIRT group with a median time from treatment to post-treatment scan of 35 days. We are not aware of any other studies of the longitudinal changes in metabolic liver function after SIRT, but based on the treatment type (radiation) it could probably be compared to SBRT, in which the nadir occurs after one month, with stabilization after three months [18]. There are no longitudinal, functional studies following RFA and TACE for comparison with the present study, but with a median time interval of 44.5 and 47 days, respectively, the patients were probably between the nadir and the stage of full regeneration. The main reason for variation in the time from treatment to scan between groups was the clinical feasibility. Aarhus University Hospital is the only center in Denmark that performs SIRT which meant that some patients had to travel. Post-treatment scans were thus combined with standard clinical examination in order to make the project feasible. 

A second limitation is the distribution of patients with cirrhosis vs. non-cirrhosis between the groups with all patients in the SIRT group having cirrhosis vs. only 12.5% (one patient) in the resection group. This distribution is closely linked to the treatment algorithm and thus represents clinical reality [21, 33]. We cannot rule out that the uneven distribution may affect our conclusions. Third, the 10-year period of the included scans, along with the use of both dynamic and static scans, adds some uncertainty to the interpretation of the results, especially for interindividual comparisons. However, the correlation between dynamic and static scans remained consistent throughout the study period. A final limitation is the relatively low number of patients in each treatment group which is due to the study being a single-center study, with Aarhus University Hospital being the only center in the world using ^18^F-FDGal PET/CT clinically. Since ^18^F-FDGal can be produced at any facility producing standard ^18^F-FDG [34] and since ^18^F-FDGal PET/CT has a high sensitivity for extrahepatic HCC [13] as well as potential for metabolic studies as shown here, we hope that other centers will start using ^18^F-FDGal PET/CT.

## Conclusions

HCCs > 3 cm in diameter contributed substantially to the liver’s total metabolic capacity when quantified by ^18^F-FDGal PET/CT, suggesting that this may also apply to other test substrates. Our study thus emphasizes the advantage of imaging-based methods compared to systemic tests such as the ICG clearance. The regenerative response in mean liver metabolism following locoregional treatment was decreased in patients with cirrhosis, and the type of locoregional treatment seemed to impact the response but this needs further investigation. The change in functional liver volume after locoregional treatment for HCC did not necessarily reflect a corresponding change in the total metabolic activity of the liver. These findings could impact clinical decision-making when choosing locoregional treatment to induce liver growth of the remnant liver before liver resection or when assessing the tolerability of further treatments in patients with recurrent HCC. More clinical studies addressing these issues are strongly encouraged.

## Electronic supplementary material

Below is the link to the electronic supplementary material.

Supplementary Material 1

## Data Availability

The data supporting the findings of this study are not openly available due to sensitivity reasons. However, they can be obtained from the corresponding author upon reasonable request, provided that permission is granted by the Central Denmark Region Committees on Health Research Ethics.

## Abbreviations

^18^F-FDGal: [^18^F]-fluoro-2-deoxy-D-galactose
%ID: Percent of Injected Dose
%ID/L: Percent of Injected Dose per Tissue Volume
AFP: Plasma Alpha-Fetoprotein
HCC: Hepatocellular Carcinoma
HCV: Hepatitis C Virus
ICG: Indocyanine Green
PET: Positron Emission Tomography
RFA: Radiofrequency Ablation
SBRT: Stereotactic Body Radiation Therapy
SIRT: Selective Internal Radiation Therapy
TACE: Transarterial Chemoembolization
VOI: Volumes of Interest
