# Supplementary material for: Evaluation of the effect of locoregional treatment on metabolic liver function in hepatocellular carcinoma using 18F-FDGal PET/CT
Source: EJNMMI Res. 2025 Jul 16;15:88. doi: 10.1186/s13550-025-01285-9 (PMC12267743; doi:10.1186/s13550-025-01285-9)
Supplement: Supplementary file 1 — Supplementary Material 1 [file 13550_2025_1285_MOESM1_ESM.docx]

**Supplementary**

Functional liver volume and mean radioactivity in the liver 10–20 min and 60 min after injection of ^18^F-FDGal

In total, 32 ^18^F-FDGal PET/CT scans with a dynamic scan, either 0–20 min or 10–20 min, and a static scan 60 min after the injection of the tracer, ^18^F-FDGal, were evaluated. Five of these scans were of patients without an ^18^F-FDGal PET scan after locoregional treatment and thus only used to evaluate the relationship between the dynamic and static scans.

The relationships between functional liver volume and mean radioactivity concentration (kBq/ml) in the liver for dynamic scans 10–20 min after injection of ^18^F-FDGal and static scans 60 min after injection of ^18^F-FDGal were evaluated with linear regression.

For the functional liver volume, a positive linear relationship was established with the functional volume (mL) in the liver 60 min after injection of ^18^F-FDGal equal to 279.0 mL + 0.833 mL x volume (mL) 10–20 min after injection of ^18^F-FDGal. This equation was used to estimate the functional liver volume (mL) 60 min after injection of ^18^F-FDGal in the 23 cases with only a dynamic scan 0–20 min after the injection of ^18^F-FDGal. R-squared (the coefficient of determination) was 0.91.

Supplementary Fig. 1. Relationship between volume (mL) 10–20 min and 60 min after injection of ^18^F-FDGal.


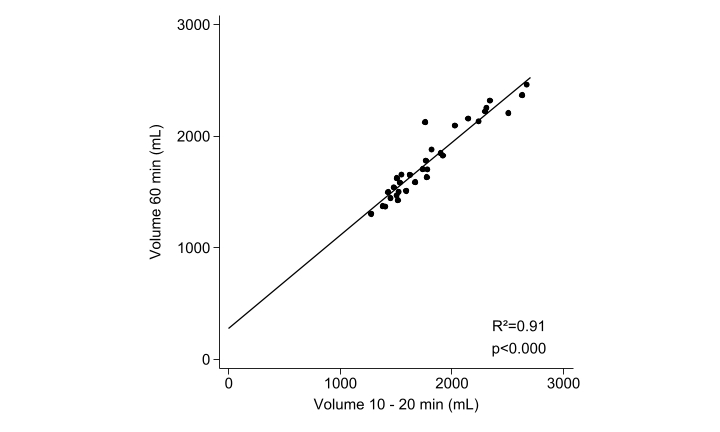


For the mean radioactivity concentration, a positive linear relationship was established with the mean radioactivity (kBq/mL) in the liver 60 min after injection of ^18^F-FDGal equal to 1.015 (SE ± 0.816) + 0.972 (SE ± 0.0508) x mean radioactivity in the liver (kBq/mL) 10–20 min after injection of ^18^F-FDGal. This equation was used to estimate the mean radioactivity (kBq/mL) 60 min after injection of ^18^F-FDGal in the 23 cases with only a dynamic scan 0–20 min after the injection of ^18^F-FDGal. R-squared (the coefficient of determination) was 0.92.

Supplementary Fig. 2. Relationship between mean radioactivity (kBq/mL) 10–20 min and 60 min after injection of ^18^F-FDGal.


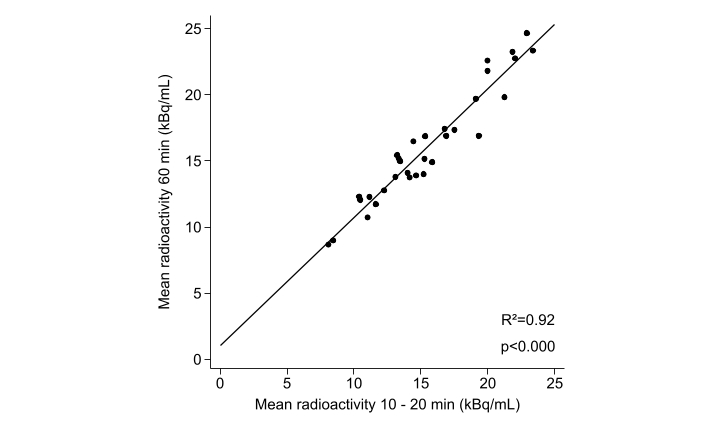


Distribution of patients with dynamic scans

In 20 of the 29 patients, the pre- and post-treatment scans were comparisons of actual data with extrapolated data. 19 of the 20 dynamic scans included were post-treatment scans.

Supplementary Table 1. Distribution of 20 included patients with dynamic scans.

| **Treatment, n (% of included patients in subgroup)** | | | |
| --- | --- | --- | --- |
| Resection | RFA | TACE | SIRT |
| 5 (63%) | 6 (75%) | 6 (67%) | 3 (75%) |
|  | | | |
| **Cirrhosis status, n (% of included patients in subgroup)** | | | |
| Cirrhosis | No cirrhosis |  |  |
| 11 (73%) | 9 (64%) |  |  |
